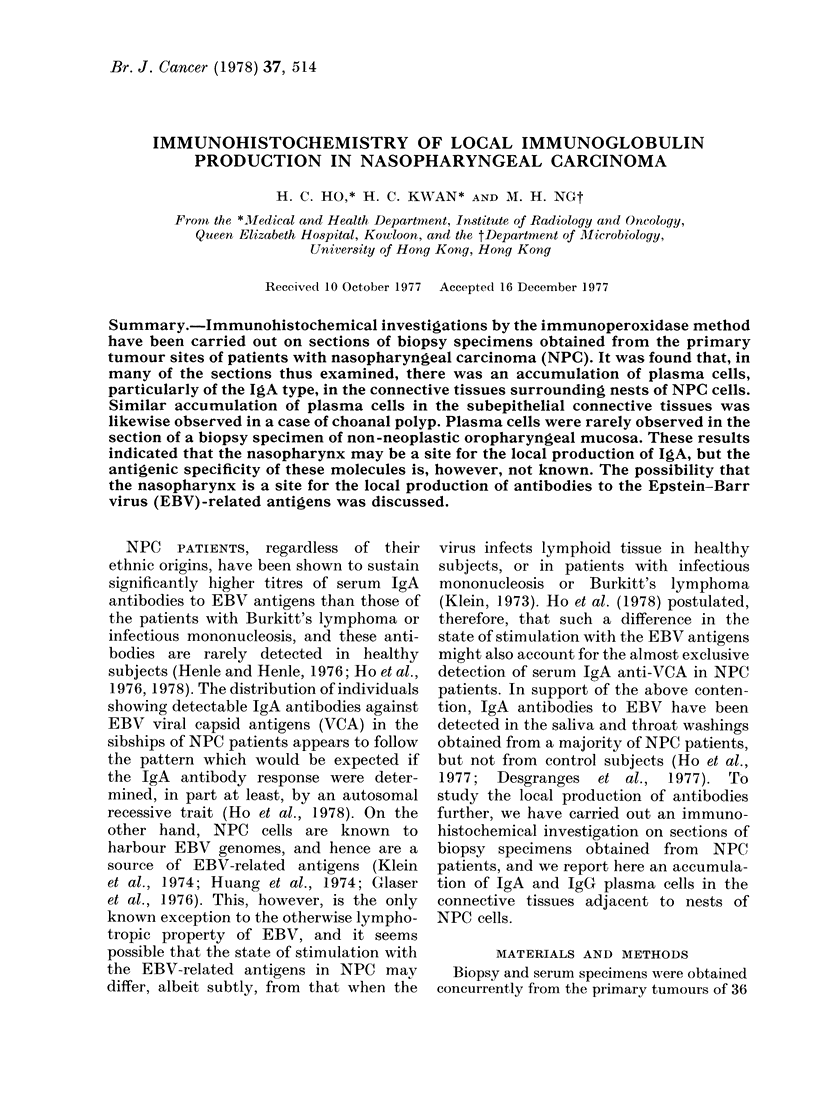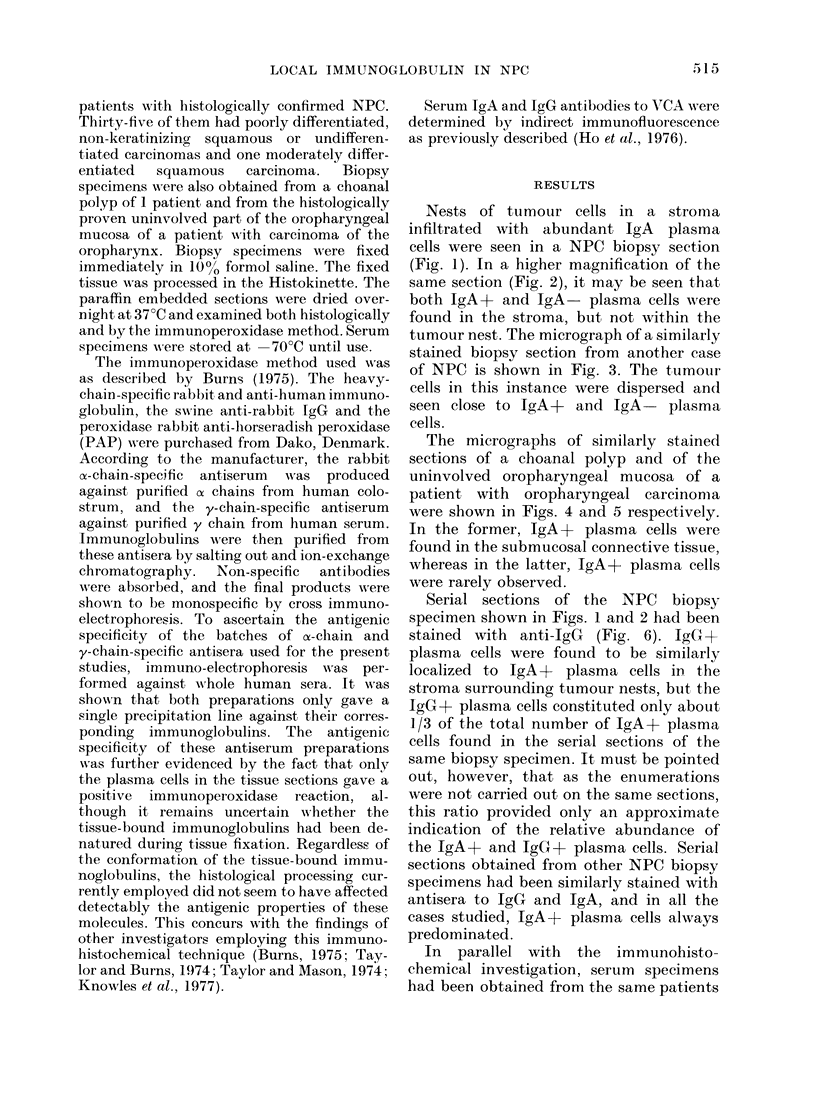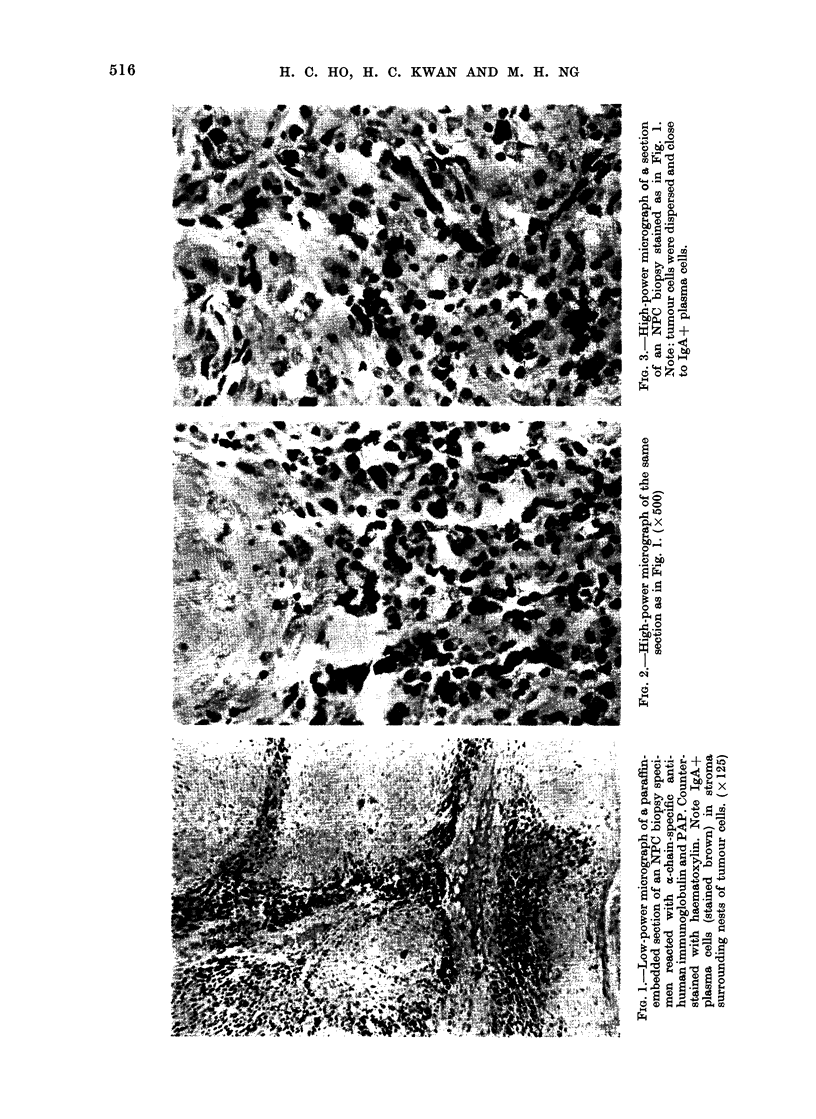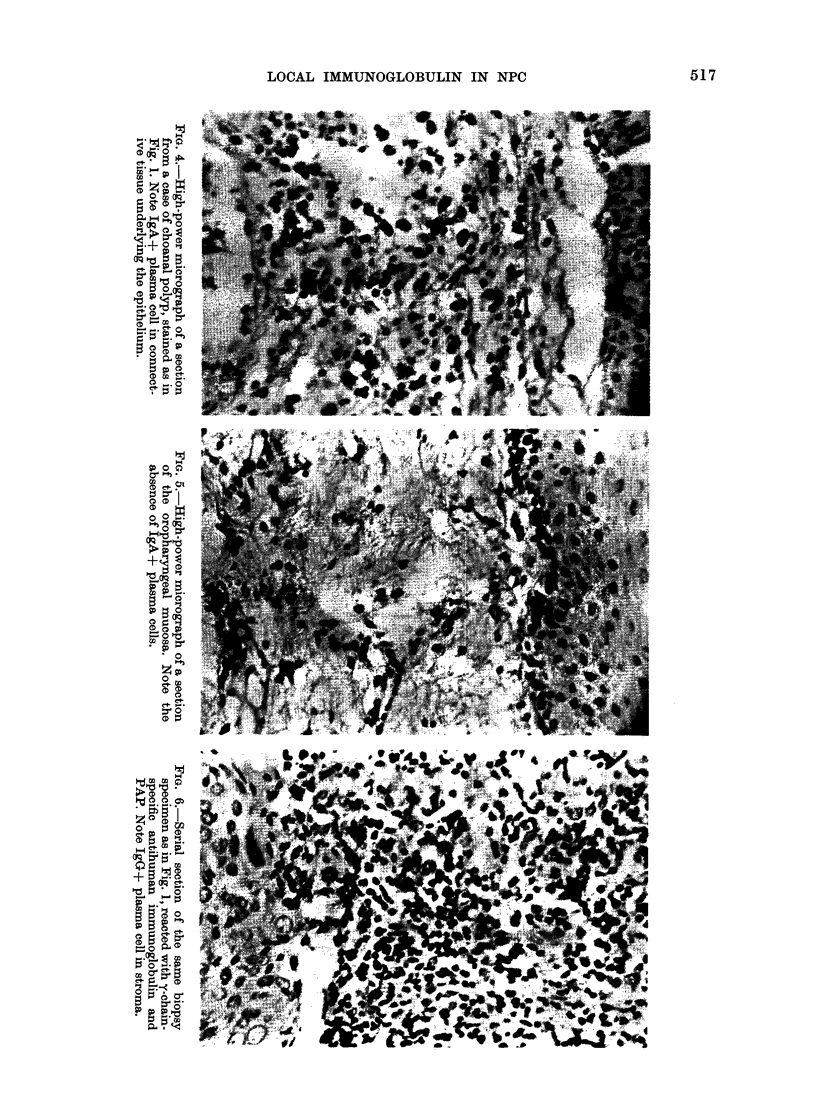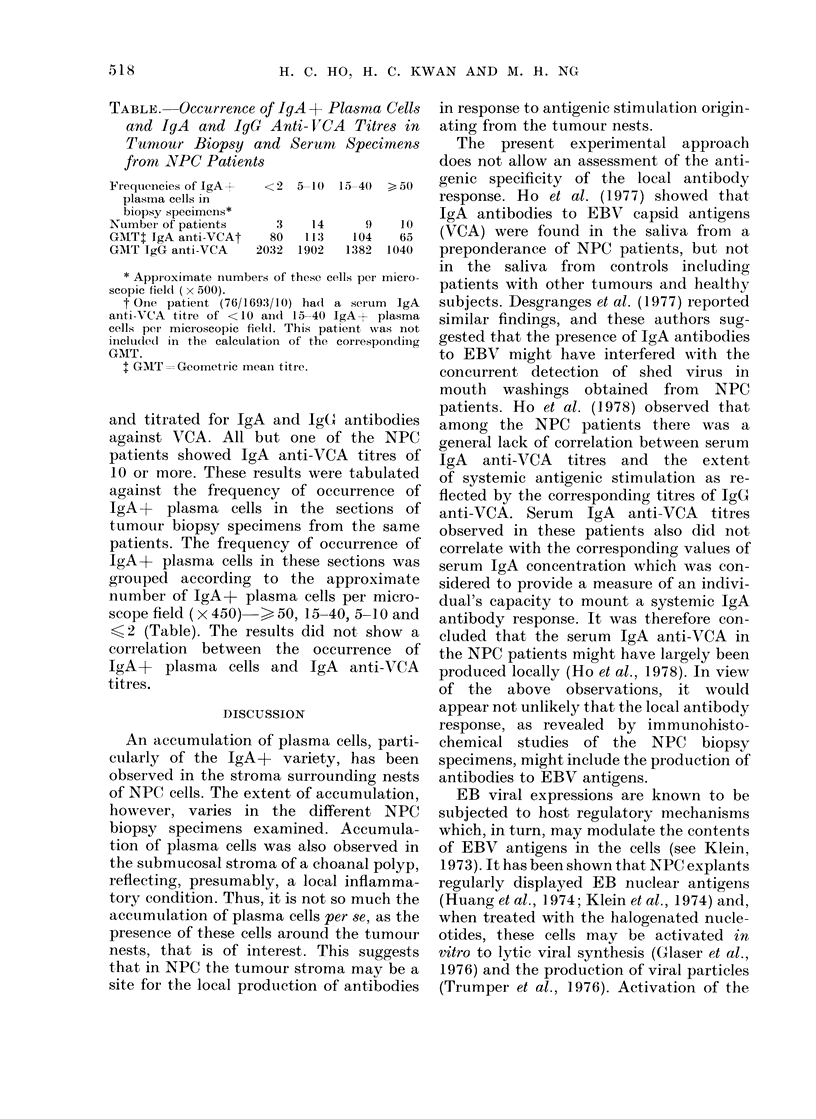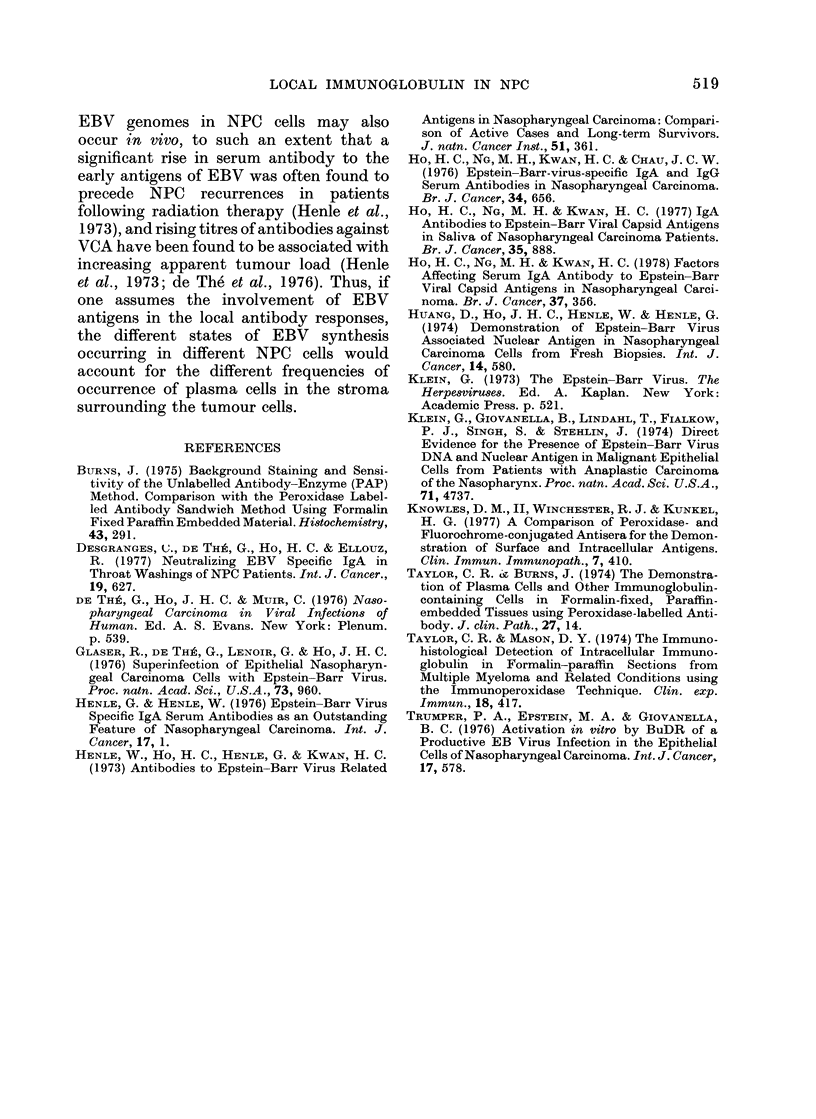# Immunohistochemistry of local immunoglobulin production in nasopharyngeal carcinoma.

**DOI:** 10.1038/bjc.1978.79

**Published:** 1978-04

**Authors:** H. C. Ho, H. C. Kwan, M. H. Ng

## Abstract

**Images:**


*Br. J. Cancer* (1978) **37**, 514

### IMMUNOHISTOCHEMISTRY OF LOCAL IMMUNOGLOBULIN PRODUCTION IN NASOPHARYNGEAL CARCINOMA

H. C. HO,* H. C. KWAN* AND M. H. NG†

*From the *Medical and Health Department, Institute of Radiology and Oncology, Queen Elizabeth Hospital, Kowloon, and the †Department of Microbiology, University of Hong Kong, Hong Kong*

Received 10 October 1977 Accepted 16 December 1977

**Summary.—Immunohistochemical investigations by the immunoperoxidase method have been carried out on sections of biopsy specimens obtained from the primary tumour sites of patients with nasopharyngeal carcinoma (NPC). It was found that, in many of the sections thus examined, there was an accumulation of plasma cells, particularly of the IgA type, in the connective tissues surrounding nests of NPC cells. Similar accumulation of plasma cells in the subepithelial connective tissues was likewise observed in a case of choanal polyp. Plasma cells were rarely observed in the section of a biopsy specimen of non-neoplastic oropharyngeal mucosa. These results indicated that the nasopharynx may be a site for the local production of IgA, but the antigenic specificity of these molecules is, however, not known. The possibility that the nasopharynx is a site for the local production of antibodies to the Epstein–Barr virus (EBV)-related antigens was discussed.**

NPC PATIENTS, regardless of their ethnic origins, have been shown to sustain significantly higher titres of serum IgA antibodies to EBV antigens than those of the patients with Burkitt's lymphoma or infectious mononucleosis, and these antibodies are rarely detected in healthy subjects (Henle and Henle, 1976; Ho *et al.*, 1976, 1978). The distribution of individuals showing detectable IgA antibodies against EBV viral capsid antigens (VCA) in the sibships of NPC patients appears to follow the pattern which would be expected if the IgA antibody response were determined, in part at least, by an autosomal recessive trait (Ho *et al.*, 1978). On the other hand, NPC cells are known to harbour EBV genomes, and hence are a source of EBV-related antigens (Klein *et al.*, 1974; Huang *et al.*, 1974; Glaser *et al.*, 1976). This, however, is the only known exception to the otherwise lymphotropic property of EBV, and it seems possible that the state of stimulation with the EBV-related antigens in NPC may differ, albeit subtly, from that when the virus infects lymphoid tissue in healthy subjects, or in patients with infectious mononucleosis or Burkitt's lymphoma (Klein, 1973). Ho *et al.* (1978) postulated, therefore, that such a difference in the state of stimulation with the EBV antigens might also account for the almost exclusive detection of serum IgA anti-VCA in NPC patients. In support of the above contention, IgA antibodies to EBV have been detected in the saliva and throat washings obtained from a majority of NPC patients, but not from control subjects (Ho *et al.*, 1977; Desgranges *et al.*, 1977). To study the local production of antibodies further, we have carried out an immunohistochemical investigation on sections of biopsy specimens obtained from NPC patients, and we report here an accumulation of IgA and IgG plasma cells in the connective tissues adjacent to nests of NPC cells.

### MATERIALS AND METHODS

Biopsy and serum specimens were obtained concurrently from the primary tumours of 36

patients with histologically confirmed NPC. Thirty-five of them had poorly differentiated, non-keratinizing squamous or undifferentiated carcinomas and one moderately differentiated squamous carcinoma. Biopsy specimens were also obtained from a choanal polyp of 1 patient and from the histologically proven uninvolved part of the oropharyngeal mucosa of a patient with carcinoma of the oropharynx. Biopsy specimens were fixed immediately in 10% formol saline. The fixed tissue was processed in the Histokinette. The paraffin embedded sections were dried overnight at 37°C and examined both histologically and by the immunoperoxidase method. Serum specimens were stored at −70°C until use.

The immunoperoxidase method used was as described by Burns (1975). The heavy-chain-specific rabbit and anti-human immunoglobulin, the swine anti-rabbit IgG and the peroxidase rabbit anti-horseradish peroxidase (PAP) were purchased from Dako, Denmark. According to the manufacturer, the rabbit $\alpha$-chain-specific antiserum was produced against purified $\alpha$ chains from human colostrum, and the $\gamma$-chain-specific antiserum against purified $\gamma$ chain from human serum. Immunoglobulins were then purified from these antisera by salting out and ion-exchange chromatography. Non-specific antibodies were absorbed, and the final products were shown to be monospecific by cross immuno-electrophoresis. To ascertain the antigenic specificity of the batches of $\alpha$-chain and $\gamma$-chain-specific antisera used for the present studies, immuno-electrophoresis was performed against whole human sera. It was shown that both preparations only gave a single precipitation line against their corresponding immunoglobulins. The antigenic specificity of these antiserum preparations was further evidenced by the fact that only the plasma cells in the tissue sections gave a positive immunoperoxidase reaction, although it remains uncertain whether the tissue-bound immunoglobulins had been denatured during tissue fixation. Regardless of the conformation of the tissue-bound immunoglobulins, the histological processing currently employed did not seem to have affected detectably the antigenic properties of these molecules. This concurs with the findings of other investigators employing this immunohistochemical technique (Burns, 1975; Taylor and Burns, 1974; Taylor and Mason, 1974; Knowles *et al.*, 1977).

Serum IgA and IgG antibodies to VCA were determined by indirect immunofluorescence as previously described (Ho *et al.*, 1976).

### RESULTS

Nests of tumour cells in a stroma infiltrated with abundant IgA plasma cells were seen in a NPC biopsy section (Fig. 1). In a higher magnification of the same section (Fig. 2), it may be seen that both IgA+ and IgA− plasma cells were found in the stroma, but not within the tumour nest. The micrograph of a similarly stained biopsy section from another case of NPC is shown in Fig. 3. The tumour cells in this instance were dispersed and seen close to IgA+ and IgA− plasma cells.

The micrographs of similarly stained sections of a choanal polyp and of the uninvolved oropharyngeal mucosa of a patient with oropharyngeal carcinoma were shown in Figs. 4 and 5 respectively. In the former, IgA+ plasma cells were found in the submucosal connective tissue, whereas in the latter, IgA+ plasma cells were rarely observed.

Serial sections of the NPC biopsy specimen shown in Figs. 1 and 2 had been stained with anti-IgG (Fig. 6). IgG+ plasma cells were found to be similarly localized to IgA+ plasma cells in the stroma surrounding tumour nests, but the IgG+ plasma cells constituted only about 1/3 of the total number of IgA+ plasma cells found in the serial sections of the same biopsy specimen. It must be pointed out, however, that as the enumerations were not carried out on the same sections, this ratio provided only an approximate indication of the relative abundance of the IgA+ and IgG+ plasma cells. Serial sections obtained from other NPC biopsy specimens had been similarly stained with antisera to IgG and IgA, and in all the cases studied, IgA+ plasma cells always predominated.

In parallel with the immunohistochemical investigation, serum specimens had been obtained from the same patients

TABLE.—*Occurrence of IgA+ Plasma Cells and IgA and IgG Anti-VCA Titres in Tumour Biopsy and Serum Specimens from NPC Patients*

| Frequencies of IgA+ plasma cells in biopsy specimens* | <2 | 5–10 | 15–40 | ≥50 |
|---|---|---|---|---|
| Number of patients | 3 | 14 | 9 | 10 |
| GMT‡ IgA anti-VCA† | 80 | 113 | 104 | 65 |
| GMT IgG anti-VCA | 2032 | 1902 | 1382 | 1040 |

* Approximate numbers of these cells per microscopic field (×500).

† One patient (76/1693/10) had a serum IgA anti-VCA titre of <10 and 15–40 IgA+ plasma cells per microscopic field. This patient was not included in the calculation of the corresponding GMT.

‡ GMT = Geometric mean titre.

and titrated for IgA and IgG antibodies against VCA. All but one of the NPC patients showed IgA anti-VCA titres of 10 or more. These results were tabulated against the frequency of occurrence of IgA+ plasma cells in the sections of tumour biopsy specimens from the same patients. The frequency of occurrence of IgA+ plasma cells in these sections was grouped according to the approximate number of IgA+ plasma cells per microscope field (×450)—≥50, 15–40, 5–10 and ≤2 (Table). The results did not show a correlation between the occurrence of IgA+ plasma cells and IgA anti-VCA titres.

### DISCUSSION

An accumulation of plasma cells, particularly of the IgA+ variety, has been observed in the stroma surrounding nests of NPC cells. The extent of accumulation, however, varies in the different NPC biopsy specimens examined. Accumulation of plasma cells was also observed in the submucosal stroma of a choanal polyp, reflecting, presumably, a local inflammatory condition. Thus, it is not so much the accumulation of plasma cells *per se*, as the presence of these cells around the tumour nests, that is of interest. This suggests that in NPC the tumour stroma may be a site for the local production of antibodies in response to antigenic stimulation originating from the tumour nests.

The present experimental approach does not allow an assessment of the antigenic specificity of the local antibody response. Ho *et al.* (1977) showed that IgA antibodies to EBV capsid antigens (VCA) were found in the saliva from a preponderance of NPC patients, but not in the saliva from controls including patients with other tumours and healthy subjects. Desgranges *et al.* (1977) reported similar findings, and these authors suggested that the presence of IgA antibodies to EBV might have interfered with the concurrent detection of shed virus in mouth washings obtained from NPC patients. Ho *et al.* (1978) observed that among the NPC patients there was a general lack of correlation between serum IgA anti-VCA titres and the extent of systemic antigenic stimulation as reflected by the corresponding titres of IgG anti-VCA. Serum IgA anti-VCA titres observed in these patients also did not correlate with the corresponding values of serum IgA concentration which was considered to provide a measure of an individual's capacity to mount a systemic IgA antibody response. It was therefore concluded that the serum IgA anti-VCA in the NPC patients might have largely been produced locally (Ho *et al.*, 1978). In view of the above observations, it would appear not unlikely that the local antibody response, as revealed by immunohistochemical studies of the NPC biopsy specimens, might include the production of antibodies to EBV antigens.

EB viral expressions are known to be subjected to host regulatory mechanisms which, in turn, may modulate the contents of EBV antigens in the cells (see Klein, 1973). It has been shown that NPC explants regularly displayed EB nuclear antigens (Huang *et al.*, 1974; Klein *et al.*, 1974) and, when treated with the halogenated nucleotides, these cells may be activated *in vitro* to lytic viral synthesis (Glaser *et al.*, 1976) and the production of viral particles (Trumper *et al.*, 1976). Activation of the

EBV genomes in NPC cells may also occur *in vivo*, to such an extent that a significant rise in serum antibody to the early antigens of EBV was often found to precede NPC recurrences in patients following radiation therapy (Henle *et al.*, 1973), and rising titres of antibodies against VCA have been found to be associated with increasing apparent tumour load (Henle *et al.*, 1973; de Thé *et al.*, 1976). Thus, if one assumes the involvement of EBV antigens in the local antibody responses, the different states of EBV synthesis occurring in different NPC cells would account for the different frequencies of occurrence of plasma cells in the stroma surrounding the tumour cells.

### REFERENCES

BURNS, J. (1975) Background Staining and Sensitivity of the Unlabelled Antibody–Enzyme (PAP) Method. Comparison with the Peroxidase Labelled Antibody Sandwich Method Using Formalin Fixed Paraffin Embedded Material. *Histochemistry*, **43**, 291.

DESGRANGES, C., DE THÉ, G., HO, H. C. & ELLOUZ, R. (1977) Neutralizing EBV Specific IgA in Throat Washings of NPC Patients. *Int. J. Cancer.*, **19**, 627.

DE THÉ, G., HO, J. H. C. & MUIR, C. (1976) *Nasopharyngeal Carcinoma in Viral Infections of Human.* Ed. A. S. Evans. New York: Plenum. p. 539.

GLASER, R., DE THÉ, G., LENOIR, G. & HO, J. H. C. (1976) Superinfection of Epithelial Nasopharyngeal Carcinoma Cells with Epstein–Barr Virus. *Proc. natn. Acad. Sci., U.S.A.*, **73**, 960.

HENLE, G. & HENLE, W. (1976) Epstein–Barr Virus Specific IgA Serum Antibodies as an Outstanding Feature of Nasopharyngeal Carcinoma. *Int. J. Cancer*, **17**, 1.

HENLE, W., HO, H. C., HENLE, G. & KWAN, H. C. (1973) Antibodies to Epstein–Barr Virus Related Antigens in Nasopharyngeal Carcinoma: Comparison of Active Cases and Long-term Survivors. *J. natn. Cancer Inst.*, **51**, 361.

HO, H. C., NG, M. H., KWAN, H. C. & CHAU, J. C. W. (1976) Epstein–Barr-virus-specific IgA and IgG Serum Antibodies in Nasopharyngeal Carcinoma. *Br. J. Cancer*, **34**, 656.

HO, H. C., NG, M. H. & KWAN, H. C. (1977) IgA Antibodies to Epstein–Barr Viral Capsid Antigens in Saliva of Nasopharyngeal Carcinoma Patients. *Br. J. Cancer*, **35**, 888.

HO, H. C., NG, M. H. & KWAN, H. C. (1978) Factors Affecting Serum IgA Antibody to Epstein–Barr Viral Capsid Antigens in Nasopharyngeal Carcinoma. *Br. J. Cancer*, **37**, 356.

HUANG, D., HO, J. H. C., HENLE, W. & HENLE, G. (1974) Demonstration of Epstein–Barr Virus Associated Nuclear Antigen in Nasopharyngeal Carcinoma Cells from Fresh Biopsies. *Int. J. Cancer*, **14**, 580.

KLEIN, G. (1973) The Epstein–Barr Virus. *The Herpesviruses.* Ed. A. Kaplan. New York: Academic Press. p. 521.

KLEIN, G., GIOVANELLA, B., LINDAHL, T., FIALKOW, P. J., SINGH, S. & STEHLIN, J. (1974) Direct Evidence for the Presence of Epstein–Barr Virus DNA and Nuclear Antigen in Malignant Epithelial Cells from Patients with Anaplastic Carcinoma of the Nasopharynx. *Proc. natn. Acad. Sci. U.S.A.*, **71**, 4737.

KNOWLES, D. M., II, WINCHESTER, R. J. & KUNKEL, H. G. (1977) A Comparison of Peroxidase- and Fluorochrome-conjugated Antisera for the Demonstration of Surface and Intracellular Antigens. *Clin. Immun. Immunopath.*, **7**, 410.

TAYLOR, C. R. & BURNS, J. (1974) The Demonstration of Plasma Cells and Other Immunoglobulin-containing Cells in Formalin-fixed, Paraffin-embedded Tissues using Peroxidase-labelled Antibody. *J. clin. Path.*, **27**, 14.

TAYLOR, C. R. & MASON, D. Y. (1974) The Immunohistological Detection of Intracellular Immunoglobulin in Formalin-paraffin Sections from Multiple Myeloma and Related Conditions using the Immunoperoxidase Technique. *Clin. exp. Immun.*, **18**, 417.

TRUMPER, P. A., EPSTEIN, M. A. & GIOVANELLA, B. C. (1976) Activation *in vitro* by BuDR of a Productive EB Virus Infection in the Epithelial Cells of Nasopharyngeal Carcinoma. *Int. J. Cancer*, **17**, 578.